# Comparison of the efficacy between continuoushemodiafiltration with polymethylmethacrylate (PMMA) membrane hemofilter CH-1.8W^®^ and with pmma membrane dialyzer BK-2.1P^®^ in the treatment of critically ill patients

**DOI:** 10.1186/2197-425X-3-S1-A62

**Published:** 2015-10-01

**Authors:** D Harada, K Matsuda, T Moriguchi, N Harii, J Goto, M Yanagisawa, H Sugawara, J Takamino, T Yoshino, Y Hasebe

**Affiliations:** Department of Emergency and Critical Care Medicine, University of Yamanashi School of Medicine, Yamanashi, Japan

## Introduction

We have reported the efficacy of continuous hemodiafiltration (CHDF) with a cytokine-adsorbing hemofilter in the treatment of hypercytokinemia, such as severe sepsis, septic shock, using a polymethylmethacrylate (PMMA) membrane dialyzer (BK-2.1P^®^; Toray Medical Co Ltd, Tokyo, Japan) [1], [2]. In 2013, a new PMMA hemofilter (CH-1.8W^®^; Toray Medical Co Ltd, Tokyo, Japan) was released in Japan. It is covered by Japanese medical insurance for continuous renal replacement therapy.

## Objectives

The aim of this study is to investigate the differences of the efficacy as cytokine-adsorbing hemofilter between BK-2.1P^®^ and CH-1.8W^®^ in the treatment of critically ill patients.

## Methods

Forty-eight consecutive patients with acute kidney injury admitted to the intensive care unit (ICU) at the University of Yamanashi Hospital and treated with CHDF in the period from July 2011 to January 2015 were entered in this study. Twenty-four consecutive patients were treated with BK-2.1P^®^ between July 2011 and August 2013 (BK2.1P group). Twenty-four consecutive patients were treated with CH-1.8W^®^ between July 2013 and January 2015 (CH1.8W group). We compared changes in systolic blood pressure, volume of urine, water balances and blood level of interleukin-6 (IL-6) for 7 days from the initiation of CHDF. In addition, we evaluated twenty-eight day and ninety day survivals. Statistical analysis was performed using the statistical software packages Prism for Mac OS X (version 6.0f).

## Results

The average ages in BK2.1P group and CH1.8W group were 63.8 ± 14.8 and 65.1 ± 15.1, respectively. The numbers of male were 16 and 17, respectively. APACHE II scores were 29.3 ± 8.8 and 26.8 ± 6.4, respectively. SOFA scores were 13.4 ± 3.3 and 12.3 ± 2.7, respectively. There were no significant differences in the pretreatment clinical profiles between the two groups. There were no significant differences of the changes in systolic blood pressure, urine volume and water balances per days following a week. At the initiation of CHDF, the blood levels of IL-6 (log) (pg/mL) were 3.61 ± 1.01 and 3.94 ± 1.08, respectively. There were no significant differences in IL-6 levels and the change ratios each day. There were also no significant differences in twenty-eight day and ninety day survivals. Ratios of the observed ninety day survivals and the predicted survivals calculated with APACHE II scores were 179.1% and 189.5%, respectively.

## Conclusions

This study suggests that there were no differences in the clinical course of critically ill patients received CHDF with CH-1.8W and with BK-2.1P. We expect that the new hemofilter contributes to improvement of the survival of the critically ill patients.
